# The impact of the COVID-19 pandemic on the decrease in the use of intensive care units in the postoperative period of anatomic lung resections. A retrospective analysis

**DOI:** 10.1590/0100-6991e-20223140-en

**Published:** 2022-06-23

**Authors:** ISMAEL RODRIGO DIAS, MARIO CLAUDIO GHEFTER, PEDRO HILTON DE ANDRADE, LILIANNE LOUISE SILVA MORAIS, MARCO AURELIO MARCHETTI, HEBERT SANTOS HIMURO, RAFAEL LACERDA PEREIRA FEICHAS

**Affiliations:** 1 - Hospital do Servidor Público Estadual de São Paulo- HSPE/IAMSPE, Departamento de Cirurgia Torácica - São Paulo - SP - Brasil; 2 - Hospital do Servidor Público Estadual de São Paulo- HSPE/IAMSPE, Departamento de Anestesiologia - São Paulo - SP - Brasil

**Keywords:** Lung Neoplasms, Thoracic Surgery, Intensive Care Unit, Postoperative Care, COVID-19, Neoplasias Pulmonares, Cirurgia Torácica, Unidade de Terapia Intensiva, Cuidados Pós-operatórios, COVID-19

## Abstract

**Objective:**

COVID-19 pandemic required optimization of hospital institutional flow, especially regarding the use of intensive care unit (ICU) beds. The aim of this study was to assess whether the individualization of the indication for postoperative recovery from pulmonary surgery in ICU beds was associated with more perioperative complications.

**Method:**

retrospective analysis of medical records of patients undergoing anatomic lung resections for cancer in a tertiary hospital. The sample was divided into: Group-I, composed of surgeries performed between March/2019 and February/2020, pre-pandemic, and Group-II, composed of surgeries performed between March/2020 and February/2021, pandemic period in Brazil. We analyzed demographic data, surgical risks, surgeries performed, postoperative complications, length of stay in the ICU and hospital stay. Preventive measures of COVID-19 were adopted in group-II.

**Results:**

43 patients were included, 20 in group-I and 23 in group-II. The groups did not show statistical differences regarding baseline demographic variables. In group-I, 80% of the patients underwent a postoperative period in the ICU, compared to 21% in group-II. There was a significant difference when comparing the average length of stay in an ICU bed (46 hours in group-I versus 14 hours in group-II - p<0.001). There was no statistical difference regarding postoperative complications (p=0.44).

**Conclusions:**

the individualization of the need for ICU use in the immediate postoperative period resulted in an improvement in the institutional care flow during the COVID-19 pandemic, in a safe way, without an increase in surgical morbidity and mortality, favoring the maintenance of essential cancer treatment.

## RESUMO

**Introdução:**

a pandemia de COVID-19 exigiu otimização dos fluxos institucionais hospitalares, especialmente quanto ao uso de leitos de unidade de terapia intensiva (UTI). O objetivo deste estudo foi avaliar se a individualização da indicação de recuperação pós-operatória de cirurgias pulmonares em leitos de UTI associou-se a mais complicações perioperatórias.

**Método:**

análise retrospectiva de prontuários dos pacientes submetidos a ressecções pulmonares anatômicas por câncer em hospital terciário. A amostra foi dividida em dois grupos: Grupo-I, composto pelas cirurgias realizadas entre março/2019 e fevereiro/2020, pré-pandemia, e Grupo-II, composto pelas cirurgias realizadas entre março/2020 e fevereiro/2021, período de pandemia no Brasil. Analisamos dados demográficos, riscos cirúrgicos, cirurgias realizadas, complicações pós-operatórias, tempo de UTI e de internação hospitalar. Foram adotadas medidas preventivas de COVID-19 no grupo-II.

**Resultados:**

foram incluídos 43 pacientes, 20 no grupo-I e 23 no grupo-II. Os grupos não apresentaram diferenças estatísticas quanto às variáveis demográficas basais. No grupo-I 80% dos pacientes fizeram pós-operatório em UTI, comparados a 21% do grupo-II. Houve diferença significativa na comparação de tempo médio de permanência em leito de UTI (46 horas no grupo-I versus 14 horas no grupo-II - p<0,001). Não houve diferença estatística quanto a complicações pós-operatórias entre grupos (p=0,44).

**Conclusões:**

a individualização da necessidade do uso de UTI no pós-operatório imediato de cirurgias pulmonares resultou em melhora no fluxo assistencial institucional durante a pandemia de COVID-19, de maneira segura, sem aumento na morbimortalidade cirúrgica, favorecendo a manutenção do tratamento oncológico essencial.

## INTRODUCTION

The COVID-19 pandemic in Brazil demanded measures from health systems to optimize care and thus minimize the length of hospital stay of patients and the movement of people in outpatient settings, offices, and diagnostic centers, to prevent contagion by the new coronavirus. The burden of health systems is worrying for cancer patients, as the reduction in the availability of diagnostic and therapeutic care can considerably impact the morbidity and mortality of this population. Currently, there is already a significant reduction in the diagnoses of oncological diseases, without, however, a reduction in their incidence, suggesting under-diagnosis in the pandemic period. It is estimated that the reception of patients in advanced disease stages for treatment of complications will increase considerably, as well as mortality^1^.

Patients with early-stage lung cancer have great benefit from surgical treatment and display a 5-year survival greater than 80%. However, as the disease progresses, survival falls drastically^2^. Therefore, and according to the guidelines of the Brazilian Thoracic Oncology Group, maintaining the surgical flow for the treatment of these patients has become essential^3^.

Due to the increased demand for Intensive Care Unit (ICU) beds for patients with COVID-19, there was a need to reduce perioperative ICU beds. To maintain oncological surgeries, some institutional measures were taken, as well as a change in routines by the surgical and anesthetic teams. Major surgeries, such as anatomical lung resections, which until then were routinely referred to the ICU in the immediate postoperative period, began to be referred according to an individualized decision that justified their need. This institutional change made it feasible to perform surgeries for treatment of lung cancer, in addition to optimizing hospital processes and costs.

This study aimed to analyze the efficacy and safety of using ward beds for patients in the immediate postoperative period of anatomical lung resections, as well as to assess the incidence of postoperative complications both in the ward and ICU. 

## METHODS

**Figure 1:**
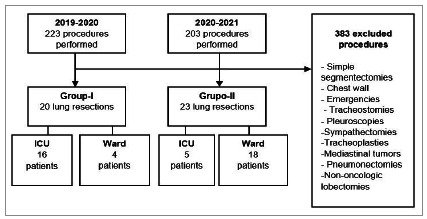
Study flowchart and eligibility criteria.

We conducted a retrospective analysis of the medical records of patients who underwent lung resections for lung cancer at the Hospital do Servidor Público Estadual de São Paulo (IAMSPE), a tertiary and teaching hospital, where the surgeries were performed by a thoracic surgery resident supervised by a staff thoracic surgeon. We analyzed surgeries carried out between March 2019 and February 2021, and we included in the study all patients undergoing lung lobectomies or anatomical segmentectomies for the treatment of lung cancer. We excluded patients undergoing other oncological surgeries, as there was no uniformity in the comparison of the two years, emergency surgeries, and non-oncological surgeries, due to the restriction in the pandemic period (Figure 1).

We divided patients into two groups: group I (G-I), operated between March 2019 and February 2020 (pre-pandemic) and group II (G-II), operated between March 2020 and February 2021 (pandemic period). All patients were evaluated preoperatively for anesthetic, cardiovascular, and pulmonary surgical risks, considering the ASA (American Society of Anesthesiologists), the Lee index, and FEV1 (as a percentage of the predicted value), respectively^4.5^. The variables analyzed were age, sex, comorbidities, preoperative risk, access route, type of resection performed, intraoperative complications, postoperative complications, need for ICU, length of ICU, and length of hospital stay.

All surgeries were performed by the same surgical team, using an anterolateral thoracotomy or video-thoracoscopy (VATS) with three ports as access routes. Pleural drainage took place in all procedures.

All patients underwent general anesthesia associated with regional anesthesia (epidural, paravertebral, or erector spinae plane block) and intraoperative maintenance of monopulmonary ventilation. In the patients included in G-I, an institutional routine recommended sending the patient to the ICU due to the availability of places at the time and considering the continuous clinical surveillance inherent to the sector as the main advantage. For G-II, however, the decision on the need for ICU was taken jointly by the surgical and anesthetic team at the end of the procedure, in an assessment individualized for each patient, considering preoperative cardiovascular and pulmonary risk criteria, intraoperative complications, and the need for clinical support at the end of surgery. Due to the pandemic, before admission, patients from G-II were instructed to strictly quarantine for 15 days and undergo an RT-PCR-COVID test, being allocated in non-COVID surgical wards. Despite the change in routine and referral of patients to the ward in the immediate postoperative period, all of them had an ICU bed reservation in case there was a need.

As for the length of hospital stay, we compared all patients referred to the ward (4 G-I and 18 G-II = 22 patients) versus those who went to the ICU (16 G-I and 5 G-II = 21 patients).

We classified surgical complications with the Clavien-Dindo scale, which defines: grade 1, as any deviation from the expected surgical course, without the need for specific pharmacological or surgical treatment, including drain time greater than 4 days; grade 2, as complications that require specific pharmacological treatment; grade 3, as complications requiring surgical, endoscopic, or radiological intervention, 3a being with local anesthesia and 3b with general anesthesia; grade 4, as life-threatening complications, 4a with single organ dysfunction and 4b with multiple organ dysfunction; and grade 5, as patient death^6^.

We compared the qualitative variables between groups I and II with the Pearson’s chi-square and Fisher’s exact tests^6^ ^,^ ^7^. For quantitative variables, we used the Mann-Whitney test to verify the difference between the means of groups I and II^8^ ^,^ ^9^. We adopted a significance level of 95%, and considered differences significant when p<0.05. The study was submitted to and approved by the Ethics in Research Committee (CEP), under number 52164021.9.0000.5463.

## RESULTS

 We included 43 patients, distributed between G-I (n=20) and G-II (n=23), with no significant differences regarding baseline characteristics, preoperative risk, and type of surgery performed. We found a significant difference regarding the access route, though (Table 1).

**Table 1:** Characteristics of patients and surgeries performed.

Variables	G-I (n=20)	G-II (n=23)	p value
Age (mean ± SD)	64.5 ± 12.06	64.6 ± 10.44	0.922
Sex			0.037
Female	5 (25%)	10 (43.4%)	
Male	15 (75%)	13 (56.6%)	
Comorbidities			
ASH	12	11	0.258
DM2	5	7	0.076
COPD/Asthma	6	10	0.486
Variables	G-I (n=20)	G-II (n=23)	p value
Cardiovascular disease	2	6	0.216
PH/PTE	1	1	1.000
CKD	1	1	1.000
None	3	3	1.000
ASA (median)	3	2	0.518
Cardiac risk - Lee			0.503
Low	9	14	
Moderate	9	7	
High	2	2	
FEV1 (%) - average	80.75%	81%	0.534
Access route			0.007
VATS	4	13	
Thoracotomy	16	10	
Surgery performed			0.456
RUL	6	6	
RLL	4	2	
ML	3	4	
Bilobectomy	1	3	
LUL	5	4	
LLL	1	3	
Anatomical segmentectomy	0	1	

SAH: Arterial Hypertension; DM 2 : Diabetes Mellitus; COPD: Chronic Obstructive Pulmonary Disease; HP: Pulmonary Hypertension; PTE: Pulmonary Thromboembolism; CKD: Chronic Kidney Disease; RUL: right upper lobectomy; LM: middle lobectomy; RLL: right lower lobectomy; LUL: left upper lobectomy LLL: lower left lobectomy.

G-I patients had four Intraoperative complications, of which three were vascular injuries requiring suture, but without serious hemodynamic repercussions, and one was a lacerated lung injury requiring suturing. In G-II, there were five intraoperative complications, of which two were bronchial injuries requiring bronchorrhaphy, one vascular injury requiring suture, without serious hemodynamic repercussions, one cardiorespiratory arrest (CPA) due to intraoperative arrhythmia, with spontaneous return of circulation after three cycles of resuscitation, and one patient with a tumor invading the a hilar vessel, requiring ligation of the intrapericardial pulmonary vein, with small bleeding in the stump and controlled with hemostatic suture. All patients were extubated in the operating room after surgery, except for the patient in G-II who had intraoperative CPA.

In G-I, 16 patients were sent to the ICU after surgery, including all who had intraoperative complications. Four patients were referred to the ward bed due to their good postoperative clinical status and the need for a bed for another critically ill patient. Of these four patients, one had a pulmonary risk considered moderate by a FEV1 of 62% and two had a moderate cardiac risk, with ASA 3; all other risks assessed were low. Among the patients in G-II, five went to the ICU, all of whom had intraoperative complications. Of these, five were ASA 3, two had pulmonary risk considered high by FEV1 55% and 57%, and one patient had moderate pulmonary risk, with a FEV1 of 75%, one had high cardiac risk and the other two had moderate cardiac risk, including the patient who sustained cardiac arrest. Two patients had low cardiac and pulmonary risks.

The mean time of ICU stay was 46 hours for G-I, with one patient staying for 16 days due to bleeding on the 2nd postoperative day, requiring urgent reintervention and evolution to pneumonia, pleural empyema, and, finally, death on the 16th postoperative day. Eight G-I patients remained in the ICU for more than 24 hours. In G-II, the mean ICU time was 14 hours, with 1 patient staying for seven days due to intraoperative CPA consequences and the need for ventilatory support for five days, and other two patients staying for more than 24 hours. The length of ICU stay showed a statistically significant difference between groups (p<0.001).

The difference between groups regarding surgical complications was not statistically significant (Table 2). In G I, we recorded 14 surgical complications (70%): 10 were grade 1 (all requiring a drain longer than 4 days), one grade 2 (surgical site infection), one grade 3b (loculated empyema), one grade 4a (PTE), and one grade 5 (death). In G II, 14 patients had some complication (60%): seven were grade 1 (drain time longer than 4 days), five grade 2 (asthma decompensation, empyema, atrial fibrillation, and pneumonia), and two grade 5 (death).

**Table 2:** Comparative analysis of outcomes.

Outcomes	G-I (n=20)	G-II (n=23)	p-value
Intraoperative complications	4 (20%)	5 (21.7%)	0.442
Clavien-Dindo Classification			0.311
Grade-1	10	7	
Grade-2	1	5	
Grade-3a	0	0	
Grade-3b	1	0	
Grade-4a	1	0	
Grade-4b	0	0	
Grade-5	1	2	
Extubation in OR	20 (100%)	22 (95.6%)	0.535
Postoperative destination			<0.001
ICU	16 (80%)	5 (21.7%)	
Ward	4 (20%)	18 (78.3%)	
ICU time in hours (mean ± SD)	46 ± 61.17	14 ± 59.0	<0.001

We recorded no cases of COVID-19 among G-II patients during hospitalization or within 15 days of outpatient return.

We observed a reduction in hospital stay, with a mean of 7.8 days (range 2 25) for patients in the immediate postoperative period in a ward bed versus 12.9 days (range 3 80) for patients in the immediate postoperative period in an ICU bed.

## DISCUSSION

Disease progression in lung cancer patients is inversely related to patient survival, so the change in institutional flow evaluated in this study was implemented to ensure the maintenance of curative surgical procedures in this population. We can see that there was no reduction in surgical volume when comparing the pre-pandemic period with the one of the first pandemic wave in Brazil.

The evaluated pre-pandemic institutional routine, of a mandatory postoperative ICU bed, was designed in a context of greater availability of beds and directed to lung resection surgeries in a population with several comorbidities, in which intensive care can help recovery from the disease^4^ ^,^ ^10^. However, our analysis showed no statistical difference in postoperative complications and morbidity and mortality when comparing patients who were referred or not for immediate recovery in the ICU when the surgical procedures did not present with any surgical or anesthetic complications.

Our findings corroborate the results of Cerfolio et al.^11^, who in a series of 500 patients undergoing pulmonary resection found a rate of 76% of patients referred to the ward and observed no significant difference in the incidence of postoperative complications.

The use of ICU beds in G-II patients only if necessary accelerated recovery and reduce exposure to risks inherent to the ICU, such as higher rates of infection and delirium, in addition to the significant impact on hospital costs and improvements in institutional processes^12^. In G-II patients, ICU bed stay was effective for clinical optimization in all cases due to individualized indication, which represents a significant improvement when compared with the rates of effective ICU use in only about 6% of cases in the postoperative period of thoracic surgeries reported in previous work^13^ ^-^ ^15^.

In the context of the COVID-19 pandemic, the institutional improvement evaluated in this study was crucial to enable cancer treatment to the analyzed population, given that the first wave lasted almost a year and then new restrictive measures were taken during the second wave, causing a further reduction in the availability of beds and greater restriction of hospital expenses. The reduction of approximately five days in hospital stay for patients referred to the ward reflects the greater efficiency of the fast-track model adopted, whose efficacy and safety have already been evaluated in a study by Schmocker on accelerated hospital discharge^12^.

Despite not being one of the institutional goals in the period evaluated, there was a change in the pattern of the surgical access route adopted, with greater performance of VATS in G-II patients. Considering that VATS reduces the risk of complications and the length of hospital stay compared with thoracotomy, this characteristic can be considered a confounding factor for the evaluated outcomes^16^ ^,^ ^17^.

The postoperative complications found in this study were mostly of a minor degree and without the need for any clinical intervention. However, there were three deaths, of which two were late and due to causes unrelated to the surgical intervention and only one occurred early on the 4^th^ PO by bilateral PTE occurred on the 3^rd^ PO of a patient who evolved well in the first two days, with scheduled chest drain removal and hospital discharge. Although this event occurred in the ward in a patient from G-II, the implementation of the London Protocol showed that the referral to the ICU in the immediate postoperative period would not have changed the patient’s clinical evolution^18^.

The main limitations of this study are the small sample size, with limited power, and its retrospective observation, which hampers establishing causal relationship or controlling confounding factors in the results. Furthermore, we did not carry out a comparative analysis of hospital costs between groups due to the unavailability of expenditure data for each patient. However, we believe that there is external validity in encouraging the decision and referral of these patients to the ICU to be objective and judicious, which can occur in any other service.

## CONCLUSION

Maintenance of surgical treatment of patients with lung cancer during the COVID-19 pandemic can be performed efficiently and safely through individualized assessment of the need for postoperative intensive care. Patients undergoing pulmonary resection surgeries without anesthetic or surgical complications and who are clinically stable benefited from the immediate postoperative period in the ward, with no evidence of increased complications.
